# Draft Genome Sequence of the Betaproteobacterial Endosymbiont Associated with the Fungus *Mortierella elongata* FMR23-6

**DOI:** 10.1128/genomeA.01272-14

**Published:** 2014-12-11

**Authors:** Reiko Fujimura, Ayumu Nishimura, Shoko Ohshima, Yoshinori Sato, Tomoyasu Nishizawa, Kenshiro Oshima, Masahira Hattori, Kazuhiko Narisawa, Hiroyuki Ohta

**Affiliations:** a Ibaraki University College of Agriculture, Ibaraki, Japan; b National Research Institute for Cultural Properties, Tokyo, Japan; c Department of Computational Biology, Graduate School of Frontier Science, the University of Tokyo, Chiba, Japan

## Abstract

The fungus *Mortierella elongata* FMR23-6 harbors an endobacterium inside its mycelium. Attempts to isolate the endobacterium from the fungus were not yet successful, but a highly purified bacterial fraction was prepared. Here, we report the draft genome sequence of the endobacterium.

## GENOME ANNOUNCEMENT

A betaproteobacterial endosymbiont was found in the mycelium of a N_2_O-producing soil fungal isolate, *Mortierella elongata* FMR23-6 (1). Although the endobacterium has yet to be isolated from the fungus, a 16S rRNA gene-based phylogenetic analysis indicated that the endobacterium was clustered with the fungal endosymbionts *Burkholderia rhizoxinica* HKI 454^T^ (2–4) and “*Candidatus* Glomeribacter gigasporarum” (5, 6) in the family *Burkholderiaceae* (1). Here, we investigated the draft genome of the endobacterium of *M. elongata* FMR23-6 to obtain information about its physiological characteristics and interactions with the host fungus.

Endobacterial DNA was extracted from the bacterial fraction of the mycelium homogenate of *M. elongata* FMR23-6. In brief, the fungal mycelium, grown on half-strength cornmeal-malt-yeast extract (CMMY) agar (1), was suspended in saline, disrupted with sterile glass beads (diameters, 2.5 to 3.5 mm), and the suspension was centrifuged (1,800 × *g* for 10 min) to remove the large mycelial debris. The supernatant was passed through 8- and 3-µm-pore membranes and further treated with DNase (TaKaRa Bio, Kyoto, Japan) to remove the fungal DNA (6). Bacterial cells were separated by centrifugation on a Nycodenz density gradient, as described by Ikeda et al. (7). DNA of the endobacterial fraction was extracted using the lysozyme buffer method (8).

The draft genome sequence of the endobacterium was obtained using the Roche 454 Titanium system, with 39-fold coverage. These 454 reads were assembled in 12 scaffolds containing 97 contigs using 454 Newbler Assembler version 2.7. Sanger sequencing (ABI 3730xl; Applied Biosystems) with PCR and primer walking was performed for gap closing and resequencing of low-quality regions in the assembled data. The total nucleotide length of the assembled genome was 2,653,566 bp, with a G+C content of 46.1%. The genome sequence was annotated automatically using the RAST server version 2.0 (9), followed by manual annotation with the NCBI-nr (http://www.ncbi.nlm.nih.gov) and Swiss-Prot (http://www.uniprot.org) databases using the BLASTP program (10). The KAAS server (11) was also used for annotation and metabolic pathway prediction using predicted open reading frame (ORF) sequences, which were obtained from RAST annotation.

The endobacterium draft genome contained one rRNA operon, 39 tRNAs, and 2,390 predicted coding sequences. The annotation results revealed that the endobacterial genome carries 119 phage-associated genes (including 27 integrase genes) and 36 transposase genes. The KAAS results indicated that the endobacterium lacks cysteine biosynthetic pathways, suggesting that the endobacterium might obtain cysteine through the host fungus. The genome contained the type III secretion system genes, which were reported to play a crucial role in bacterial infection into the fungal mycelia (12). Furthermore, genes encoding the insecticidal toxin complex protein were also found in the endobacterial genome. With respect to this finding, it seems to be of interest to note that the phytotoxin (rhizoxin) is synthesized by the endosymbiotic bacterium *B. rhizoxinica* but not by the phytopathogenic fungus *Rhizopus microsporus* itself (2). Future studies are now in progress to isolate the endobacterium from *M. elongata* FMR23-6.

### Nucleotide sequence accession numbers.

This whole-genome shotgun project has been deposited at DDBJ/EMBL/GenBank under the accession no. BBOF00000000. The version described in this paper is the first version, BBOF01000000.
